# An Atypical Presentation of Cerebellar Abscess: A Case Report

**DOI:** 10.7759/cureus.60847

**Published:** 2024-05-22

**Authors:** Priyanka Hampe, Dinesh V Hinge, Keta Vagha, Sham Lohiya, Jayant D Vagha

**Affiliations:** 1 Department of Pediatrics, Jawaharlal Nehru Medical College, Acharya Vinoba Bhave Rural Hospital, Datta Meghe Institute of Higher Education and Research, Wardha, IND

**Keywords:** hydrocephalus, neurosurgery, infratentorial abscess, dermoid cyst, congenital defect

## Abstract

An infratentorial abscess is a medical emergency. Common sources of abscesses are otogenic foci, sinusitis, or dental abscess, rarely congenital defects like dermoid cysts with sinus along with cerebrospinal axis can lead to infratentorial abscess. This case report describes a four-year-old girl with pus discharging from the occipital area. Radiological imaging revealed a cerebellar abscess with the sinus tract open exteriorly through an occipital cortical defect with obstructive hydrocephalus. The patient underwent neurosurgical intervention followed by antibiotic therapy. Histopathology of the tissue sample was suggestive of a dermoid cyst. Congenital defects should not be ignored. All newborns should have a thorough physical examination to identify birth defects. As these defects can cause life-threatening complications, early recognition with early surgical intervention is the treatment of choice.

## Introduction

A dermoid cyst is a rare, benign neoplasm. Its incidence is 0.1-0.7% in all intracranial tumours [1]. Childhood tumours associated with dermoid sinus are uncommon. Dermoid cyst was first described by Lannelongue and Achard in 1860, and surgical treatment for this condition was first described by Horrax in 1992 [2]. The dermoid cyst is usually located in the posterior fossa in the midline. In calvaria, a dermoid cyst is often present in the anterior fontanel and rarely in the occipital bone. They are sometimes associated with hemivertebrae, spina bifida, and Klippel-Feil deformity. A dermoid cyst is seen and treated by other specialists in routine practice like dermatologists, neurologists, and gynaecologists. All of them define dermoid cysts based on their experience, but the common factor is the presence of solitary or occasionally multiple hamartomatous tumours. Depending upon the location of origin, dermoid cysts contain substances like hair, bone, and cartilage, or some are just thin-walled cysts containing variable amounts of fatty tissue. They occur mainly on the face, scalp, and neck. It occurs in all age groups and is primarily present at birth, but some cysts, like ovarian dermoid cysts, can present at 15-30. Etiologically, they occur when the skin and its appendages become trapped during fetal development. Histopathologically, cysts occur as sequestration of skin along the line of closure [3].

## Case presentation

**Figure 1:**
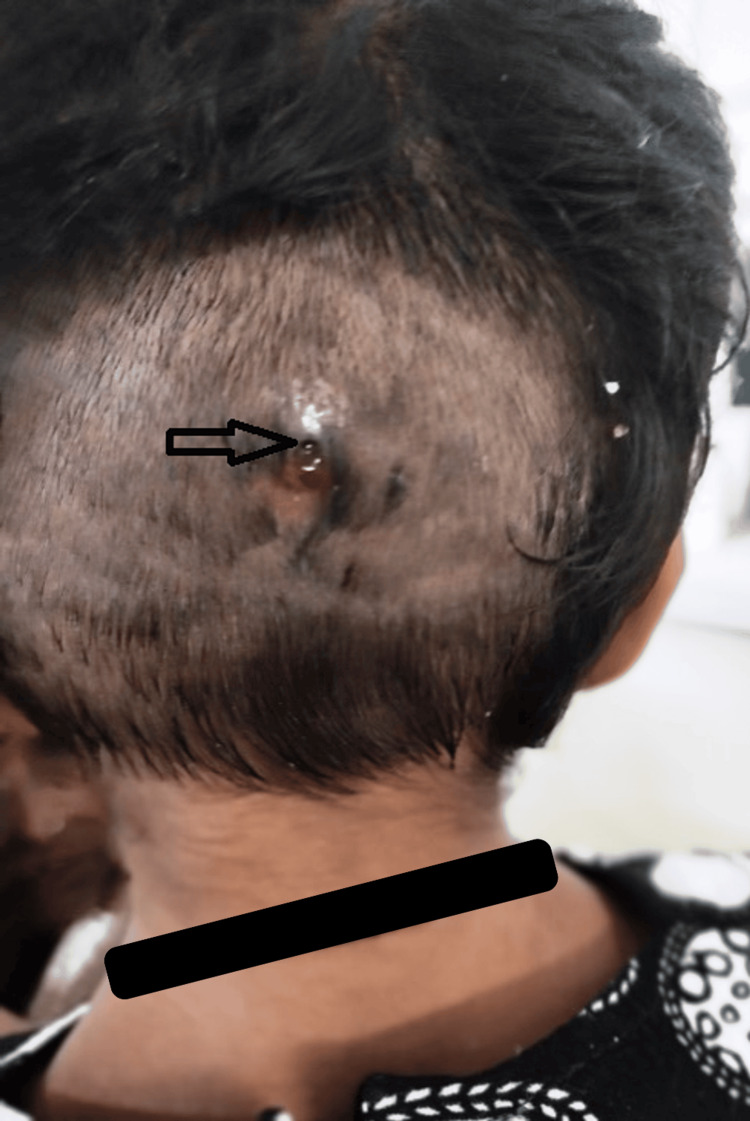
Clinical picture showing open purulent ulcer with pus discharging over the occipital area.

A four-year-old girl with normal neuropsychological development was admitted to our Institute with complaints of an open purulent ulcer with pus discharging over the occipital area for one year as shown in (Figure 1).

**Figure 2:**
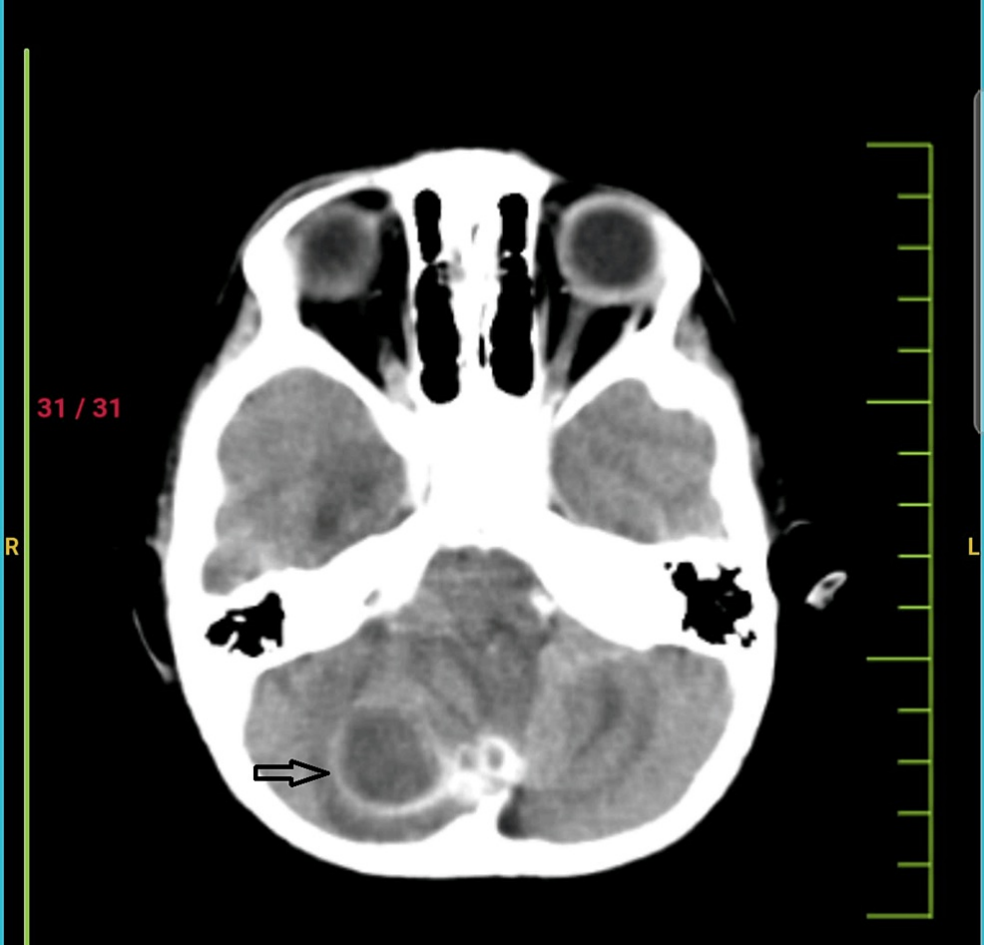
CECT showing well-defined peripherally enhancing cystic lesion. CECT: contrast-enhancing computed tomography

She had small peanut (around 1x1 cm)-sized swelling over the occipital region of the scalp since birth. Antenatal scan at 32 weeks of gestation showed small swelling over the occipital area. She was apparently well till three years of life, without any significant birth and past history in three years. Hence, parents also ignored it, as swelling neither increased nor any discharge. After three years of age, she had a high-grade fever and accidental fall, following which the swelling increased in size from around 5x5 mm to 10x10 mm with serous discharge. Incision and drainage were done. After 15 days, the child was brought to our Institute with pus discharging from the occipital scalp area. On general examination, the child was hemodynamically stable. Anthropometry wise falls on 3rd centile. on Neurological examination normal. On focused local examination, there was a small, reddish, soft swelling approximately 10x10 mm, containing a pinpoint-sized dimple in its centre at the level of the external occipital protuberance, and there was a small amount of serous discharge from the dimple. All routine investigations done like Haemogram showing neutrophilic leukocytosis, C-reactive protein was high (78 mg/dL), erythrocyte sedimentation rate was high for age (94 mm/hr), liver and renal function tests were within normal range. Radiographs of the skull anteroposterior (AP) view and lateral view were normal. On suspecting osteomyelitis or communicating sinus, contrast-enhancing computed tomography (CECT) of the brain showed or revealed a well-defined peripherally enhancing cystic altered signal intensity lesion with surrounding oedema measuring approximately 3.7x3x3 cm in the right cerebellar hemisphere (Figure 2).

**Figure 3:**
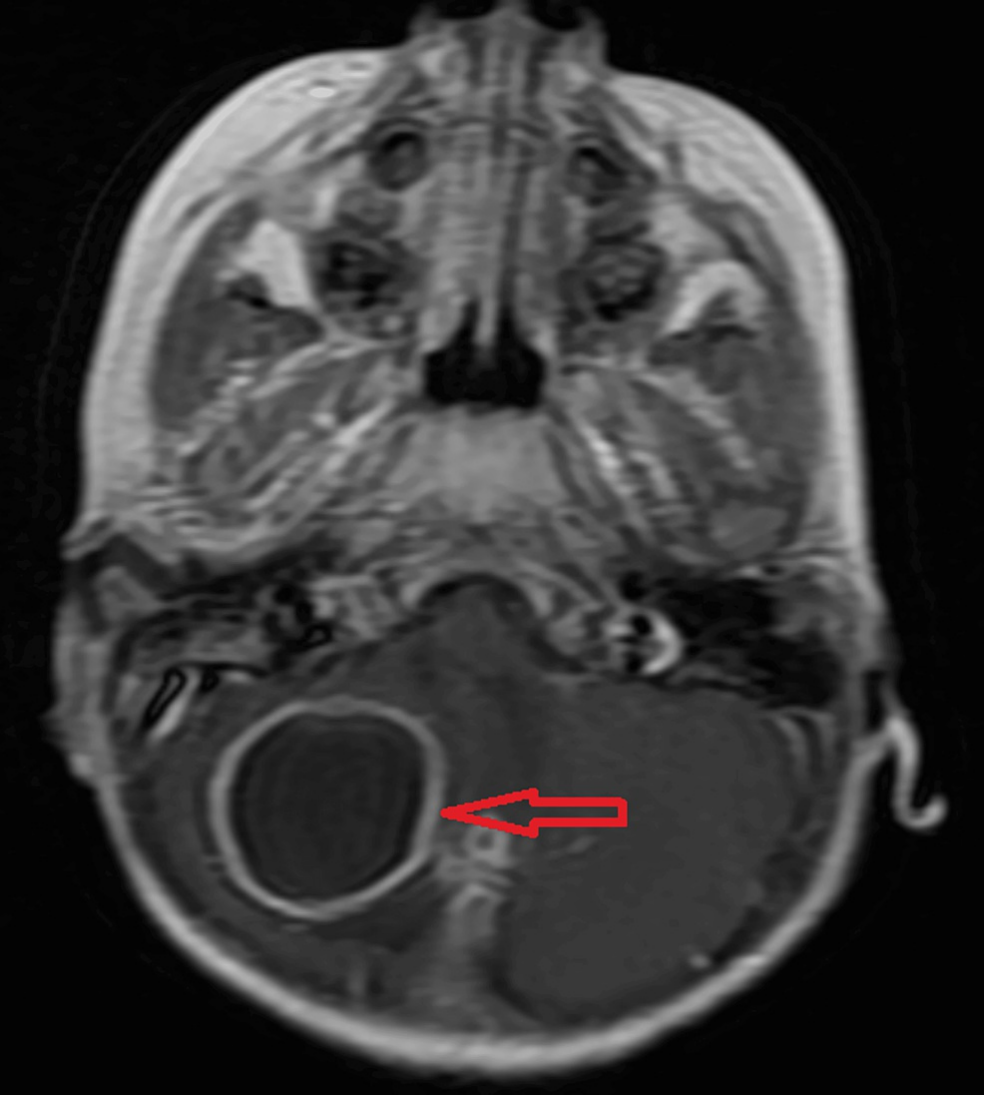
MRI brain contrast T1 weighted image showing hypo intense lesion in the right cerebellar hemisphere.

**Figure 4:**
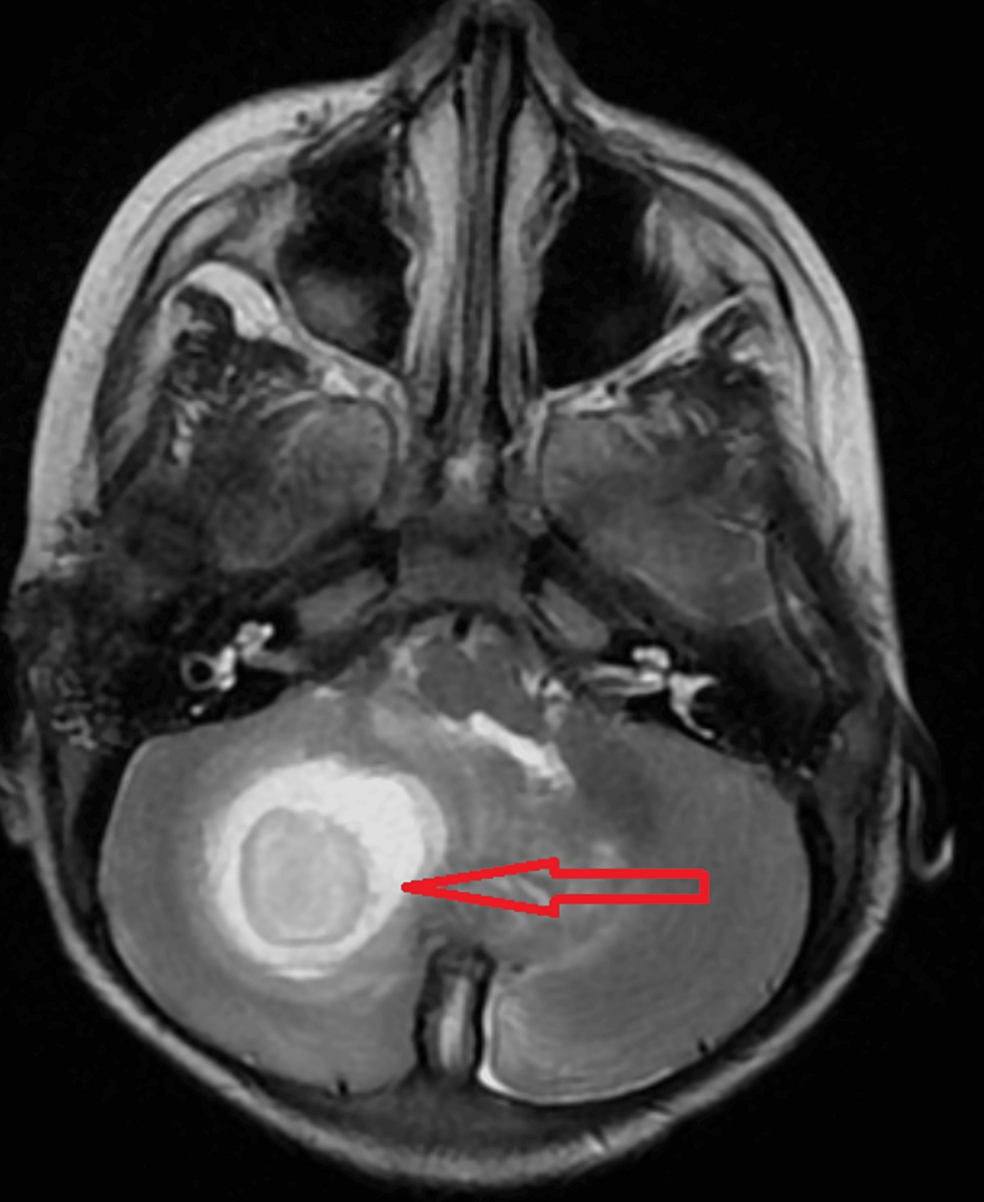
MRI brain contrast T2 weighted image showing hyperintense lesion in the right cerebellar hemisphere.

Magnetic resonance imaging (MRI) of the brain with contrast and MR spectroscopy suggested well-defined peripherally enhancing cystic altered signal intensity lesions with surrounding oedema measuring approximately 3.7x3.2x3 cm in the right cerebellar hemisphere appearing hypo intense on T1-weighted image (Figure 3) and hyperintense on T2 (Figure 4).

**Figure 5:**
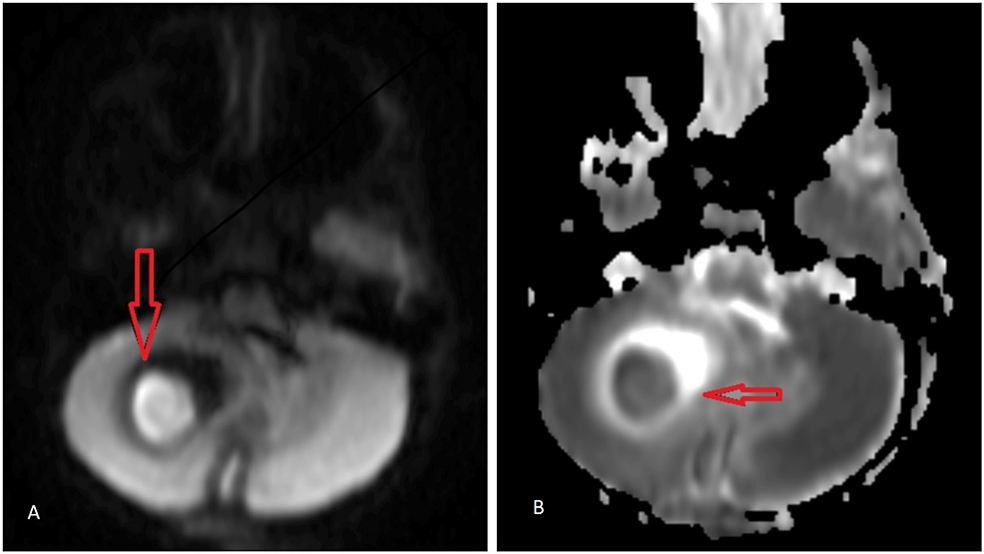
MRI brain showing (A) selective restriction on diffusion-weighted images (DWI) and (B) corresponding low signal on apparent diffusion coefficient (ADC).

MRI showing selective restriction on diffusion-weighted images (DWI) and corresponding low signal on apparent diffusion coefficient (ADC) (Figure 5).

**Figure 6:**
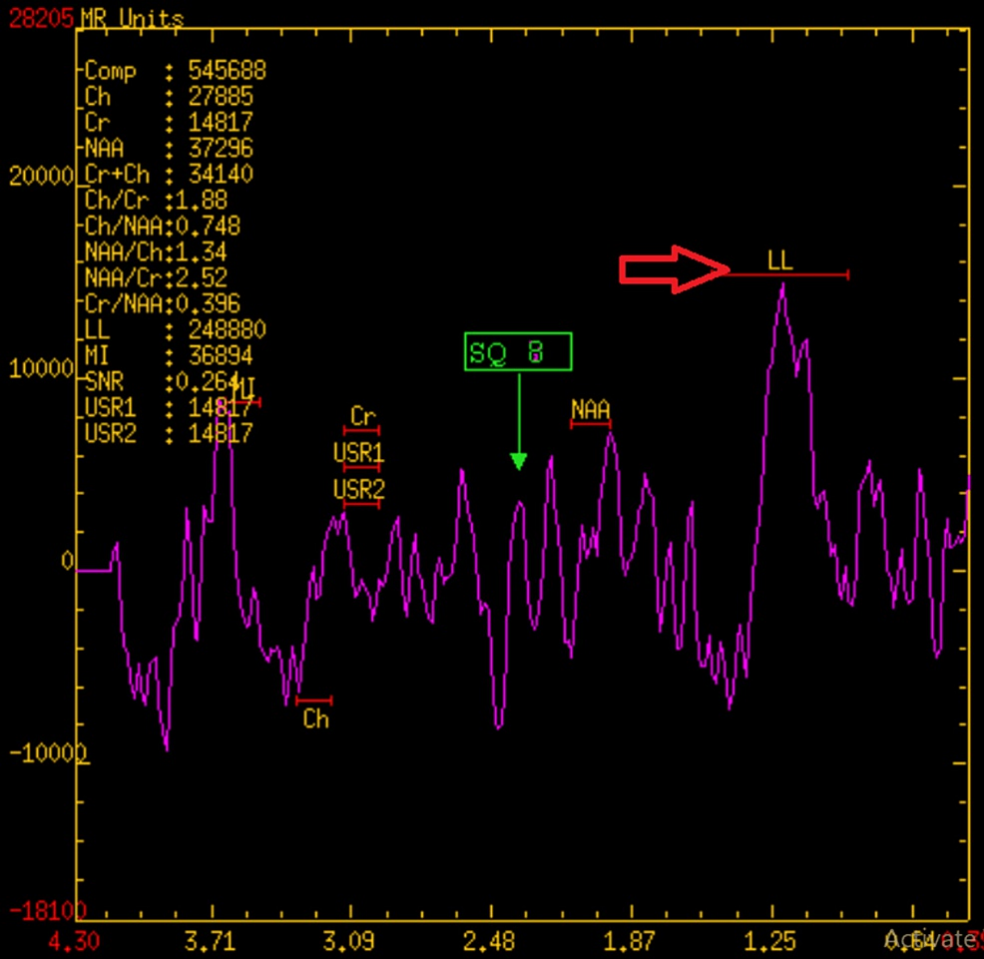
MR spectroscopy showed an increased lipid lactate peak.

Moreover, no blooming on gradient recalled echo (GRE) suggests the cerebellar abscess. The lesion caused effacement of the right cerebellar fossa, compressing the fourth ventricle, leading to dilatation of the lateral and third ventricles, leads obstructive hydrocephalous with peripherally enhancing sinus tract and opening exteriorly through a cortical defect in the midline of the occipital region. MR spectroscopy showed decreased N-acetyl-aspartate (NAA), increased choline/creatinine ratio, and increased lipid lactate, suggesting chronic inflammation (Figure 6).

**Figure 7:**
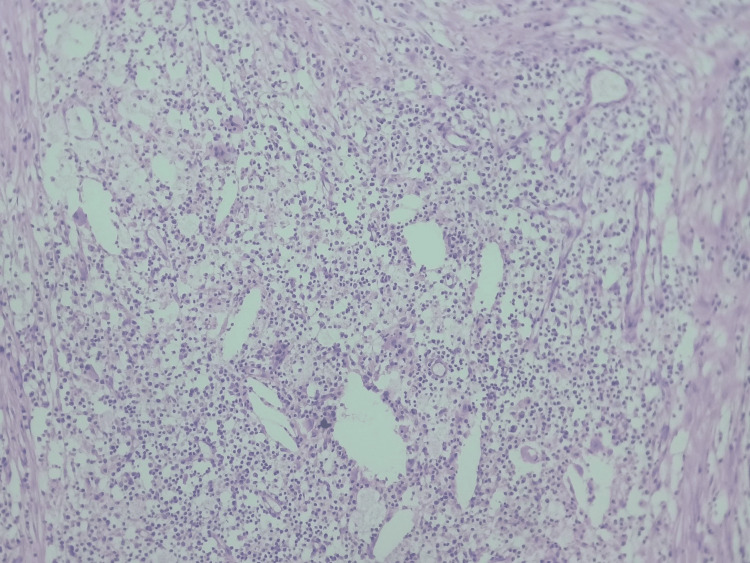
Histopathological examination was suggestive of a dermoid cyst.

The child was treated with injectable ceftriaxone and vancomycin for gram-positive and gram-negative bacteria coverage; levetiracetam was added after discussion with our unit head for prevention of convulsion and paracetamol for pain. Neurosurgical intervention was done through the paramedian incision and suboccipital craniectomy to gain access to the cerebellar abscess. Approximately 6-8 mL of pus was evacuated, followed by complete excision of the lesion with the capsule. The narrow tract lined by epithelium called dermoid sinus was also completely excised. Recurrence of posterior fossa dermoid cyst after complete resection is considered unlikely. Pus was sent for culture and antibiogram, which showed coagulase-positive *Staphylococci*. A tissue sample from the abscess wall was sent for histopathological examination and was suggestive of a dermoid cyst, as shown in (Figure 7).

Post-operative antibiotics continued for 28 days. The post-operative clinical course was uneventful except for mild restriction of neck movement for a week. After being successfully discharged, the child was followed every month and is doing well. In post-operative MRI, there was no new finding or residual abscess after one month. We have obtained written informed consent from the patient/patient’s family for the use and publication of the patient’s data and picture of the child.

## Discussion

The incidence of dermoid cysts is 0.1-0.7% among all intracranial tumours. It is usually located in a midline position. The most common site is the lumbosacral area at the cranial end. The anterior fontanelle is the most common site in calvaria, but it can rarely be present in the occipital region [4]. Longue and Till et al. classified infratentorial dermoid cysts as follows: (1) Extradural dermoid cyst with a complete sinus, (2) extradural dermoid cyst without a dermal sinus, (3) intradural dermoid cyst with a complete dermal sinus, (4) intradural dermoid cyst with an incomplete dermal sinus - only nine patients with extradural dermoid cyst arising in the posterior fossa were reported [5]. Martinez et al. reported 28 cases of posterior fossa dermoid cyst with suppuration, among which 14 cases had a cerebellar abscess and six cases had pus drainage through the sinus tract [6]. Thirty per cent of dermoid cysts have pinpoint size dimples on their surface. It is a portal of entry for microorganisms into the cerebrospinal axis. In our case, the child had pus discharge from the sinus, but the patient was asymptomatic without fever, ataxia, nystagmus, speech abnormality, or hypotonia. Most cases present with meningitis and are rarely complicated with raised intracranial pressure or seizure. We planned an X-ray of the skull AP view and lateral views. Most cases revealed oval-round, small-moderate-size defects with sclerotic margins, but in our case, no apparent changes were identified on the X-ray, probably due to the small size of the sinus.

CECT and MRI are better modalities to identify and make the correct differential diagnosis of dermoid cysts. A dermoid cyst is ectodermal in origin, and it contains an apocrine gland, sebaceous gland, eccrine gland, and lipid metabolite. CECT scan shows the cyst's exact location, size, and extent. It reveals hypodensity in the cyst. If the cyst is infected, then the density value will be higher. In the present case, a CT scan showed a well-defined hypodense lesion, i.e., right cerebellar abscess, with perilesional oedema causing obstructive hydrocephalous with linear hypodense tract, i.e., dermoid sinus opening exteriorly as a focal cranial defect. The same finding was confirmed by MRI brain contrast. A fistulogram is usually not recommended due to the risk of intracranial infection or the risk of rupture of the infected cyst. The treatment of dermoid cysts is surgical excision. Usually, there is no cyst recurrence after complete surgical excision, but Hashmi and Jones reported two cases of cerebellar abscess recurrence of cerebellar abscess 20 years later [2]. Management of a cerebellar abscess typically involves surgical intervention aimed at total evacuation of the abscess, including removal of its capsule, whenever possible.

## Conclusions

All newborns should undergo a thorough physical examination for congenital defects and, if any, be evaluated. Detailed newborn examination for midline defects and congenital anomalies like neural tube defects, anorectal malformation, or choanal atresia is recommended. A dermoid cyst is ectodermal in origin. It is congenital, but not all of them are diagnosed at birth. Early detection with timely surgical intervention is the treatment of choice to prevent the development of severe intracranial infection. Neuroimaging in the form of CECT and MRI helps to identify dermoid cysts and their associated intracranial complications. The outcome is favourable with timely surgical intervention.
